# Chronic kidney disease is common in sickle cell disease: a cross-sectional study in the Tema Metropolis, Ghana

**DOI:** 10.1186/s12882-015-0072-y

**Published:** 2015-05-29

**Authors:** Richard Kobina Dadzie Ephraim, Derick Nii Mensah Osakunor, Obed Cudjoe, Enos Amoako Oduro, Lyudmila Asante-Asamani, Juliana Mitchell, Hope Agbodzakey, Prince Adoba

**Affiliations:** Medical Laboratory Division, Department of Medical Laboratory Technology, UCC, Cape Coast, Ghana; Department of Molecular Medicine, School of Medical Sciences, Kwame Nkrumah University of Science and Technology, College of Health Sciences, Kumasi, Ghana; Sickle Cell Unit, Tema General Hospital, Tema, Ghana

**Keywords:** Chronic kidney disease, Ghana, HbSS, HbSC, Sickle cell disease

## Abstract

**Background:**

Renal involvement in sickle cell disease (SCD) contributes significantly to morbidity and mortality. The aim of this study was to determine the prevalence of chronic kidney disease (CKD) amongst SCD patients, and how basic clinical variables differ across haemoglobin genotypes.

**Methods:**

A hospital-based cross-sectional study conducted from December 2013 to May 2014 at the Sickle cell clinic of the Tema General Hospital. One hundred and ninety-four (194) participants with SCD, receiving medical care at the outpatient sickle cell clinic were enrolled onto the study. A structured questionnaire was administered to obtain information on demography, clinical history, blood pressure and anthropometry. Blood and urine samples were taken for serum creatinine and proteinuria determination respectively. The estimated GFR (eGFR) was calculated using the CKD-EPI and Schwartz equations. CKD was defined according to the Kidney Disease Improving Global Outcomes (KDIGO) guidelines. Analysis was performed using GraphPad prism and *P <*0.05 was considered statistically significant.

**Results:**

CKD was present in 39.2 % of participants. Using KDIGO guidelines, 40.8 % of the HbSS participants had stage 1 CKD and none had stage 2 CKD. In addition, 30.8 % of the HbSC participants had stage 1 CKD and 3.8 % had stage 2 CKD. There was a trend of increasing age across CKD stages and stage 2 CKD participants were oldest (P < 0.001).

**Conclusion:**

Results from the current study suggest that CKD is common amongst SCD patients and prevalence and intensity increases with age. Proteinuria and CKD was more common in HbSS genotype than in HbSC genotype.

**Electronic supplementary material:**

The online version of this article (doi:10.1186/s12882-015-0072-y) contains supplementary material, which is available to authorized users.

## Background

Sickle cell disease (SCD) is a haemoglobinopathy, which occurs due to a mutation in the globin gene of haemoglobin [1]. SCD may occur as a homozygous inheritance of haemoglobin S (HbSS), heterozygous inheritance of HbS with other β-globin mutations (e.g. HbSC) or even quantitative mutations that result in decreased or absent β-globin synthesis (the thalassaaemias) [1, 2].

Prevalence of SCD worldwide is 2 % in West Africa and in Ghana [3, 4]. SCD is characterized by red blood cell sickling, vaso-occlusion, haemolysis, acute anaemia and high morbidity and mortality and this is common in those with homozygous HbSS genotype [5]. These consequences amongst others, that result from SCD has a broad manifestation that affects various organ systems in the body [6], of which chronic kidney disease (CKD) is a part.

CKD is the presence of kidney damage or decreased kidney function, which is progressive (from three or more months to years) irrespective of clinical diagnosis [7]. Renal disease is seen in 15-18 % of all SCD patients, and is a cause of early death [8, 9]. CKD from sickle cell involves damage to multiple structures within the kidney [1]*.* The haemodynamic changes that occur with chronic anaemia, renal hypoxia that results from recurrent vaso-occlusion and haemolysis-related endothelial dysfunction can lead to functional and structural changes which may progress to CKD [8, 10-12].

In Ghana, the national neonatal sickle cell screening program is yet to cover the entire country. Hence, diagnosis of SCD in several parts of Ghana is often delayed and occurs after several visits to the hospital or clinic with acute illness. This means that organ impairment may set in long before diagnosis, increasing the risk for development of CKD and increased morbidity and mortality [3]. Furthermore, common clinical markers of renal function such as serum creatinine are not reliable indicators of early stage glomerulopathy in SCD because of the increased eGFR, lower muscle mass, and increased tubular secretion of creatinine in individuals with SCD [13, 14].

This matter is of great concern and there is dearth of information about the renal status of SCD patients in Ghana. Substantial data on the issue as well as early detection and treatment amongst this target group will be of great help. The aim of this study was to determine the prevalence of CKD amongst a population with SCD and how basic clinical variables differ across haemoglobin genotypes.

## Methods

### Study design and site

This was a hospital-based cross-sectional study with consecutive sampling technique, conducted amongst SCD patients attending the sickle cell clinic at the Tema General Hospital (TGH), Tema, Ghana. The study was conducted from December 2013 to May 2014. TGH serves as the main referral centre for residents of the south-eastern parts of Ghana and offers general and specialist care services.

### Study population

One hundred and ninety- four participants were recruited for the study. A structured questionnaire (Additional file 1) was administered to each participant via interview, to obtain information on demography and clinical history (confirmed and reviewed via patient charts). To be eligible, participants had to be aged 5 years and above with confirmed HbSS or HbSC and in a steady clinical state for at least two weeks before recruitment. Individuals with sickle cell trait (HbAS) were not included in the study. Participants with symptoms suggestive of sickle cell pain crisis, acute illness (including having a fever or needing referral to an urgent care centre), clinically suspected urinary tract infection and gross haematuria were excluded. We excluded participants who were known to be infected with HIV or with a systemic condition that could result in a glomerulopathy not related to SCA (*e.g.* active hepatitis B or C infections, systemic lupus erythematosus).

### Ethical consideration

The study was approved by the Institutional Review Board, University of Cape Coast (IRB/UCC) and the Committee of Ethics, Tema General Hospital. Participation was voluntary and written informed consent was obtained from participants or from parents and guardians of children. Data was de-identified before analysis.

### Blood pressure measurement

Trained personnel measured the blood pressure of participants (mercury sphygmomanometer and stethoscope) in accordance with recommendations of the American Heart Association [15]. Repeated measurements were taken within 5–10 minutes rest interval and the mean value was recorded as the blood pressure.

### Anthropometry

Height (to the nearest 0.1 cm) without shoes was measured with a wall-mounted ruler (LINDELS, Klippan, Sweden). Weight (to the nearest 0.1 kg) in light clothing was measured with a balance (Seca, Hamburg, Deutschland). Body Mass Index (BMI) was calculated using the formula; weight (kg)/height (m^2^). Overall obesity was defined as a BMI of ≥30 kg/m^2^, normal weight as 18.5–24.9 kg/m^2^, underweight as <18.5 kg/m^2^ and overweight as 25.0–29.9 kg/m^2^ in adults [16]. In children however, we defined according to the CDC, ≥95th percentile as obese, 85^th^-94^th^ percentiles as overweight and 5^th^-84^th^ percentile as normal and <5^th^ percentile as underweight [17,18].

### Blood sample collection and processing

Five millilitres of venous blood was obtained from each participant into serum gel separator (SST) and Ethylenediaminetetraacetic acid (EDTA) anticoagulated tubes, from which biochemical and haematological assays were performed respectively. Haematological assays were performed on fresh anticoagulated blood using an automated analyser (Mindray BC 3000 Plus, Shenzhen, China). Blood in SST’s were allowed to clot, centrifuged at 1500 g for 3 minutes, and serum creatinine was estimated using an automated chemistry analyser (Selectra Junior, Vital Scientific NV, Netherlands).

### Urine sample collection and processing

Participants provided early morning urine, collected into a clean, dry, sterile and wide-necked container. Urine protein was estimated semi-quantitatively using commercially available urine test strips (highly sensitive for albumin) as per manufacturer’s instructions. “Trace” protein is equivalent to 10 mg/100 ml or about 150 mg/24 hours (the upper limit of normal). 1+ corresponds to about 200–500 mg/24 hours; 2+ to 0.5-1.5 g/24 hours, a 3+ to 2–5 g/24 hours, and a 4+ represents 7 g/24 hours or greater.

#### Outcome criteria

The estimated GFR (eGFR) was calculated for adults using the chronic kidney disease epidemiology collaboration (CKD-EPI) equation [19] and the Schwartz equation was used for children (≤17 years) [20, 21]. The CKD-EPI equation for creatinine used was as follows:

$$ 141\times min{\left(Scr/\kappa,\;1\right)}^{\alpha}\times max{\left(Scr/\kappa,\;1\right)}^{-1.209}\times {0.993}^{Age}\times 1.018\left( if\; female\right)\times 1.159\left( if\; black\right) $$

where *Scr* is serum creatinine in mg/dl, *k* is 0.7 for females and 0.9 for males, α is −0.329 for females and −0.411 for males, min indicates the minimum of *Scr* /*κ* or 1, and max indicates the maximum of *Scr* /*κ* or 1.

The CKD-EPI equation has recently been suggested as the best option for the eGFR determination in SCD [22].

The updated Schwartz equation used was as follows:

$$ eGRF\left( ml/ min/1.73{m}^2\right)=0.413\times height(cm)/ Serum\; creatinine\left( mg/ dl\right) $$

The calculated eGFR was used to stratify the study population into stages of CKD, based on the staging system of the Kidney Disease: Improving Global Outcomes (KDIGO) guidelines for CKD. The various stages were defined as follows: Stage 1 (Kidney damage with normal or increased eGFR) = ≥90 ml/min/1.73 m^2^; Stage 2 (Kidney damage with mildly decreased eGFR) = 60–89 ml/min/1.73 m^2^; Stage 3a (mild to moderately decreased eGFR) = 45–59 ml/min/1.73 m^2^; Stage 3b (moderate to severely decreased eGFR) = 30–44 ml/min/1.73 m^2^; Stage 4 (severely decreased eGFR) = 15–29 ml/min/1.73 m^2^ and Stage 5 (Kidney failure) = <15 ml/min/1.73 m^2^ [19]. CKD according to KDIGO organization is defined as either decreased estimated glomerular filtration rate (eGFR < 60 mL/min/1.73 m^2^ corresponding to stage 3–5) or evidence of kidney damage (albuminuria, or overt proteinuria [23]. Glomerular hyperfiltration was defined as eGFR >140 [24, 25].

#### Statistical Analysis

Values are expressed as mean ± SD or frequencies and proportions. Differences between groups were determined by unpaired *t* test, Chi-square, Fisher’s exact test or ANOVA, where appropriate. A multivariate logistic regression was performed to determine the factors, which may be associated with CKD amongst different populations. *P <*0.05 was considered statistically significant. Analysis was performed using GraphPad prism version 5.0 (GraphPad software, San Diego California USA).

## Results

The results of this study showed that 39.2 % of the SCD participants had CKD. Prevalence of CKD in the paediatric population was 31.6 % and that in the adult population was 68.4 %. Glomerular hyperfiltration was present in 68.8 % of the paediatric population and present in a lower 31.2 % of the adult population.

Participants with HbSC genotype were older (30 years; *P* = 0.001) than the HbSS group (21 years) and there was a significant difference when the two groups were compared by age group (*P* = 0.035). Similarly, the HbSC group had a higher blood pressure (SBP = 114 mmHg; *P* = 0.005 and DBP = 71 mmHg; *P* = 0.011) and a higher serum creatinine (55.18 μmol/l*; P =* 0.004) than their corresponding HbSS genotype participants. Mean eGFR was however higher in the participants with HbSS genotype (136 mL/min/1.73 m^2^*)* than in those with HbSC genotype (119 mL/min/1.73 m^2^; *P* = 0.002*)*. HbSC genotype group had a higher RBC count (3.86 × 10^6^ /mm^3^; *P* = 0.000**)** and HGB concentration (14.87 g/dl; *P* = 0.011) but WBC count was higher in participants with HbSS genotype (11.83 × 10^3^/mm^3^; *P* = 0.007) (Table 1).

**Table 1:** **Baseline characteristics of study population**

Variable	Hb Genotype		Total	*P*-value
	SS	SC		
	(n = 142)	(n = 52)	(n = 194)	
Age (years)	20.77 ± 10.52	30.00 ± 13.49	23.25 ± 12.04	0.001
Gender				0.296
Male	66 (46.5)	18 (34.6)	84 (43.3)	
Female	76 (53.5)	34 (65.4)	110 (56.7)	
Age group n (%)				0.035
<10	24 (16.9)	2 (3.8)	26 (13.4)	
10-19.	48 (33.8)	10 (19.2)	58 (29.9)	
20-29	46 (32.4)	16 (30.8)	62 (32.0)	
30-39	14 (9.9)	14 (26.9)	28 (14.4)	
≥40	10 (7.0)	10 (19.2)	20 (10.3)	
Blood pressure (mmHg)				
SBP	108.65 ± 8.45	113.92 ± 6.46	110.06 ± 8.27	0.005
DBP	65.89 ± 7.67	70.65 ± 8.84	67.16 ± 8.23	0.011
BMI (Kg/m^2^)	18.48 ± 12.91	19.84 ± 3.59	18.85 ± 11.19	0.599
BMI n (%)				0.002
Underweight	106 (74.6)	20 (38.5)	126 (64.9)	
Normal	32 (22.5)	24 (46.2)	56 (28.9)	
Overweight	4 (2.8)	8 (15.4)	12 (6.2)	
*Proteinuria*				0.881
Negative	84 (59.2)	34 (65.4)	118 (60.8)	
Trace	36 (25.4)	14 (26.9)	50 (25.8)	
1+	8 (5.6)	2 (3.8)	10 (5.2)	
2+	12 (8.5)	2 (3.8)	14 (7.2)	
3+	2 (1.4)	0 (0.0)	2 (1.0)	
Serum Creatinine (μmol/l)	46.19 ± 13.39	55.18 ± 12.72	48.60 ± 13.75	0.004
eGFR, mL/min/1.73 m^2^	136.09 ± 24.70	119.19 ± 16.39	131.56 ± 23.90	0.002
Haematological profile				
WBC x 10^3^/mm^3^	11.83 ± 2.94	9.70 ± 4.31	11.26 ± 3.47	0.007
RBC x10^6^/mm^3^	2.83 ± 0.77	3.86 ± 1.10	3.11 ± 0.98	0.000
HGB (g/dl)	8.18 ± 1.45	14.87 ± 12.86	9.97 ± 11.61	0.011
HCT (%)	25.53 ± 12.98	28.46 ± 7.53	26.31 ± 19.20	0.508
PLT x 10^3^/mm^3^	318.14 ± 131.94	209.92 ± 79.58	289.13 ± 129.09	0.331

P <0.05 is significant, SBP = Systolic Blood Pressure, DBP = Diastolic Blood Pressure, BMI = Body Mass Index, eGFR = estimated Glomerular Filtration Rate, WBC = White Blood Cell, RBC = Red Blood Cell, HGB/Hb = Haemoglobin, HCT = Haematocrit, PLT = Platelet

Amongst those with CKD, HbSC participants were older (*P* <0.001) and with higher SBP (*P* = 0.005) and DBP (P = 0.011) than their HbSS counterparts. Prevalence of overweight was higher in the HbSC (22.2 %; *P* = 0.018) participants than in the HbSS (0.0 %) participants. Haematological profile showed that HbSC participants had a higher RBC count (4.15 × 10^6^ /mm^3^; *P* = 0.001) and HGB concentration (10.83 g/dl; *P* = 0.001), whereas WBC (11.43 × 10^3^/mm^3^; *P* = 0.020) and platelets (348.1 × 10^3^/mm^3^; *P* = 0.007) were higher in HbSS genotype (Table 2).

**Table 2:** **Characteristics of SCD patients with chronic kidney disease**

Variable	Hb Genotype		
	SS	SC	P-value
	(n = 58)	(n = 18)	
Age (years)	25.10 ± 9.73	35.33 ± 14.82	<0.001
Gender			
Male	32 (55.2)	4 (22.2)	0.084
Female	26 (44.8)	14 (77.8)	
Age group n (%)			
<10	2 (3.4)	0 (0.0)	0.373
10-19.	20 (34.5)	2 (11.1)	
20-29	22 (37.9)	6 (33.3)	
30-39	8 (13.8)	4 (22.2)	
≥40	6 (10.3)	6 (33.3)	
Blood pressure (mmHg)			
SBP	109.21 ± 7.04	115.78 ± 7.45	0.005
DBP	64.93 ± 7.64	74.22 ± 8.69	0.011
BMI (Kg/m^2^)	17.75 ± 3.32	19.20 ± 4.35	0.599
BMI n (%)			
Underweight	36 (62.1)	12 (66.7)	0.018
Normal	22 (37.9)	2 (11.1)	
Overweight	0 (0.0)	4 (22.2)	
Haematological profile			
WBC x 10^3^/mm^3^	11.43 ± 3.07	8.41 ± 3.82	0.020
RBC x10^6^/mm^3^	2.93 ± 0.86	4.15 ± 1.02	0.001
HGB (g/dl)	8.35 ± 1.60	10.83 ± 2.56	0.001
HCT (%)	29.74 ± 8.02	29.91 ± 7.83	0.989
PLT x 10^3^/mm^3^	348.1 ± 140.5	205.9 ± 84.42	0.007

P <0.05 is significant, SBP = Systolic Blood Pressure, DBP = Diastolic Blood Pressure, BMI = Body Mass Index, eGFR = estimated Glomerular Filtration Rate, WBC = White Blood Cell, RBC = Red Blood Cell, HGB/Hb = Haemoglobin, HCT = Haematocrit, PLT = Platelet

In Table 3, the prevalence of albuminuria, classification by eGFR and stages of CKD is shown according to SCD genotype. Using KDIGO guidelines, 39.2 % of participants had CKD. Based on haemoglobin genotype, 40.8 % of the HbSS participants had stage 1 CKD and none had stage 2 CKD. In addition, 30.8 % of the HbSC participants had stage 1 CKD and 3.8 % had stage 2 CKD.

**Table 3:** **Prevalence of albuminuria and chronic kidney disease stratified by SCD genotype**

Variable	Total number (n = 194)	Hb Genotype		*P*-value
		SS (n = 142)	SC (n = 52)	
Albuminuria	76 (39.2)	58 (40.8)	18 (34.6)	0.578
eGFR, n (%)				
≥90 mL/min/1.73 m^2^	190 (97.9)	140 (98.6)	50 (96.2)	0.454
60-89 mL/min/1.73 m^2^	4 (2.1)	2 (1.4)	2 (3.8)	
CKD, n (%)				
Stage 1: eGFR ≥ 90 + albuminuria	74 (38.1)	58 (40.8)	16 (30.8)	0.366
Stage 2: eGFR 60–89 + albuminuria	2 (1.1)	0 (0.0)	2 (3.8)	0.097
Total CKD, n (%)				
Stages (1–2)	76 (39.2)	58 (40.8)	18 (34.6)	0.578

*P* <0.05 is significant, eGFR = estimated Glomerular Filtration Rate, CKD = Chronic Kidney Disease. Hb = Haemoglobin

Baseline characteristics are further stratified by CKD status in Table 4. Participants differed only by age, with CKD participants older (28 years, *P* = 0.004) than participants with no CKD (20 years).

**Table 4:** **Baseline characteristics of SCD patients based on chronic kidney disease status**

Variable	CKD STATUS		
	CKD	No CKD	P-value
	(n = 76)	(n = 118)	
Age (years)	27.53 ± 11.77	20.49 ± 11.48	0.004
Gender			0.516
Male	36 (47.4)	48 (40.7)	
Female	40 (52.6)	70 (59.3)	
Age group n (%)			0.099
<10	2 (2.6)	24 (20.3)	
10-19.	22 (28.9)	36 (30.5)	
20-29	28 (36.8)	34 (28.8)	
30-39	12 (15.8)	16 (13.6)	
≥40	12 (15.8)	8 (6.8)	
Blood pressure			
SBP	110.76 ± 7.59	109.61 ± 8.72	0.506
DBP	67.13 ± 8.75	67.19 ± 7.96	0.975
BMI (Kg/m^2^)	18.04 ± 3.59	19.38 ± 14.09	0.560
BMI n (%)			0.871
Underweight	48 (63.2)	78 (66.1)	
Normal	24 (31.6)	32 (27.1)	
Overweight	4 (5.3)	8 (6.8)	
Haematological profile			
WBC x 10^3^/mm^3^	10.71 ± 3.46	11.62 ± 3.46	0.213
RBC x10^6^/mm^3^	3.22 ± 1.03	3.03 ± 0.95	0.366
HGB (g/dl)	8.94 ± 2.12	10.63 ± 4.80	0.487
HCT (%)	29.78 ± 9.81	24.08 ± 5.50	0.154
PLT x 10^3^/mm^3^	314.42 ± 142.26	272.85 ± 118.25	0.122

P <0.05 is significant, CKD = Chronic Kidney Disease, SBP = Systolic Blood Pressure, DBP = Diastolic Blood Pressure, BMI = Body Mass Index, eGFR = estimated Glomerular Filtration Rate, WBC = White Blood Cell, RBC = Red Blood Cell, HGB = Haemoglobin, HCT = Haematocrit, PLT = Platelet

In Table 5, the factors associated with the risk of developing CKD amongst SCD patients is shown. In the model, age remained a significant factor to the development of CKD.

**Table 5:** **Factors associated with chronic kidney disease in SCD**

Variable	OR (95 % CI)	P-value
Age (years)	0.92 (0.87-0.97)	0.001
Hb Genotype	2.11 (0.69-6.45)	0.193
Gender	1.61 (0.64-4.01)	0.310
SBP (mmHg)	0.96 (0.90-1.04)	0.321
DBP (mmHg)	1.01 (0.97-1.13)	0.204
BMI (kg/m^2^)	1.08 (0.93-1.25)	0.337

OR = odds ratio; CI = Confidence interval, Hb = Haemoglobin, SBP = Systolic Blood Pressure, DBP = Diastolic Blood Pressure, BMI = Body Mass Index

## Discussion

The haemodynamic changes that occur with chronic anaemia, renal hypoxia that results from recurrent vaso-occlusion and haemolysis-related endothelial dysfunction can lead to functional and structural changes which may progress to CKD [8, 10-12]. The results of this study showed that 39.2 % of SCD participants had CKD. Prevalence of CKD in the paediatric population was 31.6 % and that in the adult population was 68.4 %. Proteinuria and CKD was more common in HbSS genotype than in HbSC genotype. CKD is common amongst SCD patients and prevalence and intensity increases with age.

The prevalence rate of CKD observed in children is higher than that reported in other studies [1, 26, 27]. In a recent study conducted in children, a CKD prevalence of 26.5 % was reported [1], which is much lower than that observed in the present study. The prevalence of CKD observed in the adult population was higher than that reported in other studies. In a study where an adult cohort of SCD patients were followed up, a baseline CKD of 28.6 % was reported [26]. The differences in prevalence has however been suggested to be due to differences in the type of equations used across different studies [27]. The different population groups used across different studies could also account for this difference, as seen in the present study. As such, prevalence may differ within different age groups.

The prevalence of CKD in the adult cohort study however increased to 41.8 % after a five year follow up [26]. This is consistent with the high prevalence as well as the increase in prevalence with age, as observed in the present study. Of note, this study adds to evidence from other studies conducted on renal abnormalities in SCD, that CKD is common in such patients [26, 28, 29]. The trend of increase in CKD with age supports the hypothesis that, sickle cell nephropathy is a progressive condition that begins during childhood [1].

It has been suggested however, that children with sickle cell anaemia (SCA) develop renal haemodynamic alterations like renal hyperperfusion and glomerular hyperfiltration (as observed in the present study), a result of renal vasodilation associated with chronic anaemia. These changes may be followed by glomerular proteinuria and progressive renal insufficiency [27]. These haemodynamically-mediated changes have been documented histologically in patients with SCA as glomerular hypertrophy [5]. With the evidence discussed above, we can speculate that anaemia *per se,* could cause glomerular damage and our observation of a higher prevalence of albuminuria and CKD in HbSS disease (with a usually lower haemoglobin) than in other sickling haemoglobinopathies is in support of this. Much more needs to be studied on the pathogenesis of glomerular damage in SCD.

We observed a significantly lower blood pressure amongst HbSS participants. Although not significant a similar study showed a slightly higher DBP in both males and females with HbSC compared to HbSS [30]. Patients with HbSS disease have a significantly lower DBP (*P* <0.001) and slightly lower SBP (*P* = 0.09) when compared to healthy controls [31]. Furthermore, compared to normal controls, blood pressure has been shown to be lower in patients with sickle cell anaemia (SCA) [32, 33] a more common occurrence amongst HbSS individuals. Guasch *et al*., [27] proposed that in a majority of HbSS patients, systemic blood pressure does not increase in renal insufficiency when compared to non-HbSS sickling disorders. The exact mechanism that mediates this relative resistance to hypertension in patients with HbSS and renal insufficiency, they say, is not known [27]. The activation of the Nitric oxide (NO) system has been suggested to implicate the resistance to hypertension in such individuals. In other experiments, humans with SCA have been shown to excrete a higher volume of urinary Nitric oxide (NO) metabolites than in non-anemic control subjects [34], and the peripheral vessels show a decreased response to blockade of the NO system [35].

### Limitations

Due to limited resources, we were unable to use more sensitive and reliable quantitative methods of detecting proteinuria. Urine creatinine levels were not determined, although it would be of interest to perform statistical analysis according to Albumin-Creatinine Ratio (ACR) levels. Moreover, a single urine measurement was used to assess CKD based on staging criteria. Data on the availability of patients who received anti-hypertensive treatment was not available to us. There is limitation on the generalization of the study findings due to consecutive sampling technique used.

## Conclusion

Results from this study suggest that CKD is common in SCD patients. CKD prevalence and intensity increases with age in SCD patients and is more prevalent amongst adults. Proteinuria and CKD was more common in HbSS genotype than HbSC genotype. With the improvement in care and longevity of SCD patients, the high prevalence of CKD in SCD is indicative of the fact that SCD patients may be at risk for the development of progressive renal insufficiency and later renal failure.

## Additional files

Additional file 1:
**QUESTIONNAIRE.**

## Abbreviations

BMI: Body Mass Index
CKD-EPI: Chronic kidney disease epidemiology collaboration
CKD: Chronic kidney disease
eGFR: estimated Glomerular filtration rate
KDIGO: Kidney Disease Improving Global Outcomes
SCD: Sickle cell disease

## References

[1] McPherson Yee M, Jabbar SF, Osunkwo I, Clement L, Lane PA, Eckman JR, Guasch A (2011). Chronic kidney disease and albuminuria in children with sickle cell disease. Clin J Am Soc Nephrol.

[2] Powars, Elliot-Mills DD, Chan L, Hiti AL, Opas LM, Johnson C (1991). Chronic renal failure in sickle cell disease: Risk factors, clinical course, and mortality. Ann Intern Med.

[3] Ohene-Frempong K, Oduro J, Tetteh H, Nkrumah F (2008). Screening newborns for sickle cell disease in Ghana. Paediatrics.

[4] Konotey-Ahulu FID (1991). The Sickle Cell Disease Patient London and Basingstoke: Macmillan Publsihing Company.

[5] Falk RJ, Scheinman J, Phillips G, Orringer E, Johnson A, Jennette JC (1992). Prevalence and pathologic features of sickle cell nephropathy and response to inhibition of angiotensin-converting enzyme. N Engl J Med.

[6] Platt RS, Brambilla DJ, Rosse WF, Milner PF, Castro O, Steinberg MH, Klug PP (1994). Mortality in sickle cell disease. N Engl J Med.

[7] Levey AS, Stevens LA, Schmid CH, Zhang YL, Castro AF, Feldman HI, Kusek JW, Eggers P, Van Lente F, Greene T (2009). A new equation to estimate glomerular filtration rate. Ann Intern Med.

[8] Serjeant GR, Serjeant BE (2001). Sickle Cell Disease, 3rd ed. edn.

[9] Saborio P, Scheinman JI (1999). Sickle cell nephropathy. J Am Soc Nephrol.

[10] Kadiri S: Chronic kidney disease: Sickle cell nephropathy as a likely cause. Annals of Ibadan Postgraduate Med 2006;4:7-10.

[11] Day TG, Drasar ER, Fulford T, Sharpe CC, Thein SL (2012). Association between hemolysis and albuminuria in adults with sickle cell anemia. Haematologica.

[12] Haymann JP, Stankovic K, Levy P, Avellino V, Tharaux PL, Letavernier E, Grateau G, Baud L, Girot R, Lionnet F (2010). Glomerular hyperfiltration in adult sickle cell anemia: a frequent hemolysis associated feature. Clin J Am Soc Nephrol.

[13] Allon M (1990). Renal abnormalities in sickle cell disease. Arch Intern Med.

[14] Schmitt F, Martinez F, Brillet G, Giatras I, Choukroun G, Girot R, Bachir D, Galacteros F, Lacour B, Grunfeld JP (1998). Early glomerular dysfunction in patients with sickle cell anemia. Am J Kidney Dis.

[15] Kirkendall WM, Burton AC, Epstein FH, Freis ED (1967). Recommendations for human blood pressure determination by sphygmomanometers. Circulation.

[16] Adult BMI [http://www.cdc.gov/healthyweight/assessing/bmi/adult_bmi/index.html]

[17] Gonzalez-Casanova I, Sarmiento OL, Gazmararian JA, Cunningham SA, Martorell R, Pratt M, Stein AD (2013). Comparing three body mass index classification systems to assess overweight and obesity in children and adolescents. Rev Panam Salud Publica.

[18] BMI for children and teens [http://www.cdc.gov/healthyweight/assessing/bmi/childrens_bmi/about_childrens_bmi.html]

[19] Kidney Disease: Improving Global Outcomes (KDIGO) CKD Work Group (2013). KDIGO 2012 Clinical Practice Guideline for the Evaluation and Management of Chronic Kidney Disease. Kidney interSuppl.

[20] Schwartz GJ, Brion LP, Spitzer A (1987). The use of plasma creatinine concentration for estimating glomerular filtration rate in infants, children, and adolescents. Pediatric Clinics of North America.

[21] Schwartz GJ, Munoz A, Schneider MF, Mak RH, Kaskel F, Warady BA, Furth SL (2009). New equations to estimate GFR in children with CKD. Am Soc Nephrol.

[22] Arlet JB, Ribeil JA, Chatellier G, Eladari D, De Seigneux S, Souberbielle JC, Friedlander G, de Montalembert M, Pouchot J, Prie D (2012). Determination of the best method to estimate glomerular filtration rate from serum creatinine in adult patients with sickle cell disease: a prospective observational cohort study. BMC Nephrology.

[23] Stevens PE, Levin A (2012). Evaluation and management of chronic kidney disease: synopsis of the kidney disease: improving global outcomes 2012 clinical practice guideline. Ann Intern Med.

[24] Chaiken RL, Eckert-Norton M, Bard M, Banerji MA, Palmisano J, Sachimechi I, Lebovitz HE (1998). Hyperfiltration in African-American patients with type 2 diabetes. Cross-sectional and longitudinal data. Diabetes care.

[25] Pruijm M, Wuerzner G, Maillard M, Bovet P, Renaud C, Bochud M, Burnier M (2010). Glomerular hyperfiltration and increased proximal sodium reabsorption in subjects with type 2 diabetes or impaired fasting glucose in a population of the African region. Nephrol Dial Transplant.

[26] Gosmanova EO, Zaidi S, Wan JY, Adams-Graves PE (2014). Prevalence and progression of chronic kidney disease in adult patients with sickle cell disease. J Investig Med.

[27] Guasch A, Navarrete J, Nass K, Zayas CF (2006). Glomerular involvement in adults with sickle cell hemoglobinopathies: Prevalence and clinical correlates of progressive renal failure. J Am Soc Nephrol.

[28] Bodas P, Huang A, O'Riordan MA, Sedor JR, Dell KM (2013). The prevalence of hypertension and abnormal kidney function in children with sickle cell disease -a cross sectional review. BMC Nephrology.

[29] Bolarinwa RA, Akinlade KS, Kuti MA, Olawale OO, Akinola NO (2012). Renal disease in adult Nigerians with sickle cell anemia: a report of prevalence, clinical features and risk factors. Saudi J Kidney Dis Transpl.

[30] Homi J, Homi-Levee L, Gentles S, Thomas P, Serjeant G (1993). Adolescent blood pressure in a cohort study of sickle cell disease. Arch Intern Med.

[31] Thompson J, Reid M, Hambleton I, Serjeant GR (2007). Albuminuria and renal function in homozygous sickle cell disease: observations from a cohort study. Arch Intern Med.

[32] Gladwin MT, Sachdev V, Jison ML, Shizukuda Y, Plehn JF, Minter K, Brown B, Coles WA, Nichols JS, Ernst I (2004). Pulmonary hypertension as a risk factor for death in patients with sickle cell disease. N Engl J Med.

[33] Jaja SI, Opesanwo O, Mojiminiyi FB, Kehinde MO (2000). Lung function, haemoglobin and irreversibly sickled cells in sickle cell patients. West Afr J Med.

[34] Nahavandi M, Wyche MQ, Perlin E, Tavakkoli F, Castro O (2000). Nitric Oxide Metabolites in Sickle Cell Anemia Patients after Oral Administration of Hydroxyurea; Hemoglobinopathy. Hematology.

[35] Belhassen L, Pelle G, Sediame S, Bachir D, Carville C, Bucherer C, Lacombe C, Galacteros F, Adnot S (2001). Endothelial dysfunction in patients with sickle cell disease is related to selective impairment of shear stress-mediated vasodilation. Blood.

