# Supplementary material for: Chronic kidney disease is common in sickle cell disease: a cross-sectional study in the Tema Metropolis, Ghana
Source: BMC Nephrol. 2015 May 29;16:75. doi: 10.1186/s12882-015-0072-y (PMC4448314; doi:10.1186/s12882-015-0072-y)
Supplement: Additional file 1: — QUESTIONNAIRE. [file 12882_2015_72_MOESM1_ESM.pdf]

QUESTIONNAIRE

Chronic Kidney Disease in Sickle Cell Disease: a cross-sectional study in the Tema Metropolis, Ghana

This questionnaire is aimed at gathering additional informtion to determine the prevalence of chronic kidney disease amongst sickle cell patients. After reading the participation information leaflet, kindly sign the consent form preceeding the filling of this questionnaire.You will be guided through the questionnaire and this will take about 15 minutes to complete. Information gathered and published will not be linked to you in any form.Thank you for participating.

CODE:___________ **Contact:_____________** **Date: __ /__ / 20____**

Written informed consent obtained	☐

**1.0**	**Demographic Information (*please tick or write where appropriate)***				
**1.1**	**Name**				
**1.2**	**Age**	*Please write here* ………………………………………………			
**1.3**	**Gender**	Male ☐	Female ☐		
**2.0**	**Anthropometric/Clinical Data**				
	**Parameter**	**Entry**		**Parameter**	**Entry**
	Weight (cm)			SBP (mmHg)	
	Height (cm)			DBP (mmHg)	
	BMI				
**3.0**	**Miscellaneous (to be filled by interviewer)**				
**3.1**	Urine sample rovided	☐			
**3.2**	Blood samples taken	☐			
**3.3**	Hb genotype (confirm from patient records)	☐			
